# Melodic multi-feature paradigm reveals auditory profiles in music-sound encoding

**DOI:** 10.3389/fnhum.2014.00496

**Published:** 2014-07-07

**Authors:** Mari Tervaniemi, Minna Huotilainen, Elvira Brattico

**Affiliations:** ^1^Cognitive Brain Research Unit, Institute of Behavioural Sciences, University of HelsinkiHelsinki, Finland; ^2^Finnish Institute of Occupational HealthHelsinki, Finland; ^3^Brain and Mind Laboratory, Department of Biomedical Engineering and Computational Science (BECS), Aalto University School of ScienceEspoo, Finland

**Keywords:** musical expertise, auditory event-related potentials (ERPs), mismatch negativity (MMN), P3a, learning, memory, involuntary attention

## Abstract

Musical expertise modulates preattentive neural sound discrimination. However, this evidence up to great extent originates from paradigms using very simple stimulation. Here we use a novel melody paradigm (revealing the auditory profile for six sound parameters in parallel) to compare memory-related mismatch negativity (MMN) and attention-related P3a responses recorded from non-musicians and Finnish Folk musicians. MMN emerged in both groups of participants for all sound changes (except for rhythmic changes in non-musicians). In Folk musicians, the MMN was enlarged for mistuned sounds when compared with non-musicians. This is taken to reflect their familiarity with pitch information which is in key position in Finnish folk music when compared with e.g., rhythmic information. The MMN was followed by P3a after timbre changes, rhythm changes, and melody transposition. The MMN and P3a topographies differentiated the groups for all sound changes. Thus, the melody paradigm offers a fast and cost-effective means for determining the auditory profile for music-sound encoding and also, importantly, for probing the effects of musical expertise on it.

## Introduction

Intensive music making modulates brain function and structure quite dramatically. By comparing adult individuals with training in music with those individuals who lack personal experience in music making, various functional and anatomical differences have been observed (for reviews, see Jäncke, 2009; Tervaniemi, 2009, 2012; Pantev and Herholz, 2011). In the auditory modality, these findings indicate enhanced functions in cortical (e.g., Koelsch et al., 1999; Pantev et al., 2001; Fujioka et al., 2004; Tervaniemi et al., 2005; Kühnis et al., 2013) and subcortical (Musacchia et al., 2007; Wong et al., 2007; Lee et al., 2009; Strait et al., 2009) brain regions.

In the current context, several relevant findings have been obtained by investigating how brain function can be adaptively adjusted to perceive and encode musical information even when the sounds are not in the focus of the selective attention of the participants. Such a setting is particularly well suited for studies on musicians since the differences in motivational background or attentional abilities of the participants are less likely to contaminate the observations (Kujala et al., 2007). More specifically, by recording the mismatch negativity (MMN) component of the auditory event-related potentials (ERPs), we can determine the accuracy of the auditory cortex to map the regularities of the sound stream. The MMN is an index for the acoustical or cognitive incongruity between various relatively constant sound events (Näätänen et al., 2001). If the incongruity between the most plausible sound and the encountered one is large, then the MMN can be followed by a P3a response (Escera et al., 2000; Friedman et al., 2001). Traditionally, it is assumed to reflect the involuntary attention shift towards the sound input. More recently, it has been suggested to reflect the multi-stage process of sound evaluation which, in turn, leads into attention shift (Schröger et al., 2013).

Originally, the MMN was first observed and extensively investigated in an oddball paradigm, consisting of two sounds with different acoustical features (Näätänen, 1992). In other words, within a sequence of frequent standard sounds (e.g., probability of occurrence 0.9) infrequent deviant sounds are presented (e.g., with a probability of 0.1). They differ from the standard sounds, for instance, in their frequency or in duration. However, more recently, researchers have taken special efforts to render the stimulus paradigms more ecologically valid to avoid artificially simplified paradigms. A more ecologically valid stimulation paradigm in ERP research can, for instance, consist of short transposed melodies with different contour or interval structure (Tervaniemi et al., 2001; Fujioka et al., 2004; Seppänen et al., 2007), of transposed chords differing in their mode (major/minor) (Virtala et al., 2011, 2012, 2013), or of melodies composed for the experiment (Brattico et al., 2006).

However, oddball paradigms were quite time-consuming. For instance, to record brain responses to out-of-key and out-of-scale stimuli subjects an EEG session of almost 1 h was required (Brattico et al., 2006). Most recently, MMN has been studied using a so-called multi-feature paradigm in which deviant sounds with different acoustic deviances alternate with standard sounds, allowing recordings of several MMNs as fast as in 15 min (Näätänen et al., 2004; Pakarinen et al., 2007; Partanen et al., 2011; Vuust et al., 2011; Torppa et al., 2012). In that paradigm, each standard sound is followed by a different deviant, sound sequence being, for instance, Standard Deviant-Frequency Standard Deviant-Intensity Standard Deviant-Duration etc. These sound sequences can consist of sinusoidal sound complexes (Näätänen et al., 2004; Pakarinen et al., 2007), phonemes (Partanen et al., 2011), or alternating piano tones (Vuust et al., 2011; Torppa et al., 2012).

During the past 15 years, the MMN and P3a responses have been found to differentiate musicians and non-musicians in various acoustical and musical features such as pitch (Koelsch et al., 1999; Brattico et al., 2001, 2009; Fujioka et al., 2004; Marie et al., 2012), spatial sound origin (Nager et al., 2003; Tervaniemi et al., 2006), sound omission (Rüsseler et al., 2001), speech sound features (Kühnis et al., 2013), rhythm (Vuust et al., 2005), and grouping (van Zuijen et al., 2004, 2005). In all these cases, the MMN and/or P3a have been either larger and/or faster in musicians than in non-musicians, this being considered as an index of facilitated neural processing in musicians.

However, the majority of the studies on musical expertise are suboptimal for two reasons. First, in these studies the sound stimulation has lacked ecological validity. To have a full control upon the sounds and their time of occurrence, they have been presented one by one or as brief sound patterns. Spectrally, they have also been relatively simple (e.g., sinusoidal tones or chords composed of sinusoidal tones). Even the two multi-feature paradigms utilized to study music-related brain functions have not improved much in terms of ecological validity due to their repetitive characteristics of the sound sequences. Thus, compared to real music with its multitude of overlapping and temporally successive sounds, the sound environment in the former ERP experiments of music-related brain functions has been oversimplified. Furthermore, the experiments have also been quite long and thus quite demanding to some groups of participants, particularly to children.

Second, in vast majority of the studies in the field of musical expertise, the participating musicians have had training in Western classical music (for exceptions, see Vuust et al., 2005, 2012; Tervaniemi et al., 2006; Brattico et al., 2013). Investigating brain basis of musical expertise in classical musicians was an excellent starting point for the pioneering studies on musical expertise and its brain basis. Musicians with classical orientation have relatively well documented history in their training (e.g., amount of hours spent in practicing) and most of them also have started their playing quite early in age (e.g., before the age of 7). Thus it has been feasible and fruitful to start tracing the neuroplastic effects of musical expertise from classically trained musicians. One should note, however, that in our current society, not all musical activities occur in the formal training contexts of classical music. Neither do all musicians have a musical history since their childhood. For instance, many teenagers all over the world play in bands, some of them even ending up to work and earn their livings as musicians and as other professionals in the music industry, without any formal training.

In the current paper, we wish to meet these two challenges at once. Here we use a novel melodic multi-feature paradigm, introduced by Putkinen et al. (2014). It is fast in practice and has better ecological validity than previous MMN paradigms (see below). Furthermore, because of the diversity of the current musical scene, we broaden the selection of the musicians under neuroscientific investigation. For us, of specific interest was to investigate folk musicians in Finland who, in most cases, have their first training in Western classical music but who later on turn to highly original forms of music making. In most cases, the musical score is not very much utilized which makes the performance include a different cognitive load within the auditory modality. Additionally Finnish folk musicians usually perform with many instruments, either within one instrumental family (e.g., different types of flutes) or from several families (e.g., flutes, percussions, string instruments with and/or without bowing with divergent tuning systems). Thus, from the viewpoints of music perception, cognition, and performance, the demands set by their music are rather different from the classical orientation. As the first evidence about their advanced neurocognitive processes, we recently found that the early right anterior negativity (ERAN; triggered by chord incongruities among Western cadences) (Koelsch, 2009) is both quantitatively and qualitatively modulated in Finnish folk musicians compared to previous data from musicians and non-musicians (Brattico et al., 2013). More specifically, ERAN was generally larger in amplitude in folk musicians compared with non-musicians. Importantly, folk musicians showed a subsequent P3a to the strongly-violating ending chord and their late ERAN to mildly-violating ending tones was more prominent in folk musicians than in non-musicians.

**Figure 1:**
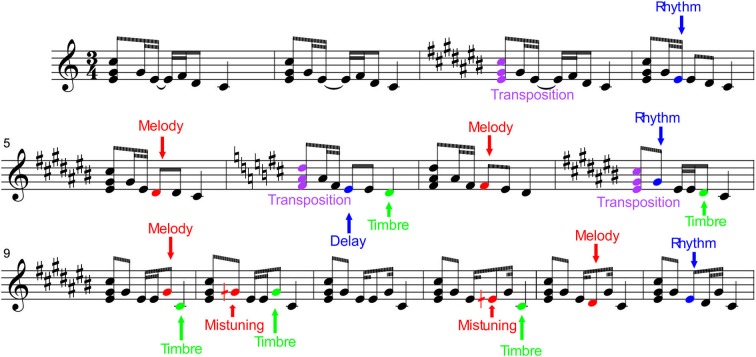
**Stimulation paradigm.** The melodies were presented while the participants were watching a silenced movie. The upward arrows indicate the changes in tuning (mistuning), timbre and timing (low-level changes which did not modulate the melody). The downward arrows indicate the changes in rhythm, melody, and key of the melody (transposition) (high-level changes which modulated the continuation of the melody). It was of specific interest to determine whether folk musicians would differ from non-musicians in all of these musical features or selectively in some of them (pitch/timbre/harmony/timing). In particular, we hypothesized that folk musicians would show superior automatic neural discrimination of low- and high-level features related to pitch, such as mistuning, as a result of the emphasis given in Finnish folk music to the melodic aspects over rhythmic ones (for details on Finnish folk music, see Saha, 1996; Asplund et al., 2006; Brattico et al., 2013). This would indicate that the specific features of each music genre can have their imprints in the neural architecture of the musicians.

Here, we investigated the auditory profile of Finnish Folk musicians using a novel melodic multi-feature paradigm lasting less than 20 min (see Figure 1). To upgrade the traditions of the ERP paradigms, it did not have any pauses even between subsequent melody presentations but instead the melody was presented in a looped manner. As introduced below, it had several kinds of deviant sounds in terms of pitch, timbre, harmony, and timing, ranging from low-level changes not affecting the melody content to high-level changes modulating the course of the successive melody. It was also transposed to 12 keys to prevent fatigue, as well as to increase sound variation and hence ecological validity.

## Methods

The experiment was approved by the Ethical committee of the Faculty of Behavioural Sciences, University of Helsinki. All participants signed an informed consent form and the study was conducted according to the principles of the Declaration of Helsinki.

### Stimuli

The stimuli consisted of brief melodies composed by the second author. They are described in detail in Putkinen et al. (2014) and briefly introduced in the following.

The sound parameters were selected on the basis of careful listening of different combinations of deviances followed by pilot EEG recordings. In the melodies, as the standard timbre, digital piano tones (McGill University Master Samples) were used to form a short melody pattern that was in accordance with Western tonal rules and was recursively repeated. Short melodies always started with a triad (300 ms), which was followed by four tones of varying length and an ending tone. There was a 50-ms gap between successive tones. The ending tone was 575 ms in duration. There was also a 125 ms gap between each melody. So, one melody lasted for 2100 ms. The melody was presented in total for 15 min, in a looped manner.

Six different deviant events (changes) were included in the melodies. They are divided into low-level changes which do not change the melody and into high-level changes which alter the melodic contour. One melody could contain several changes. For illustration, see Figure 1.

Low-level changes

1. *Mistuning* (half of a semitone upwards, 3% of the fundamental frequency of the sound) could occur in the first, second or fourth tone within the melody in 14% of the melodies.
2. *Timbre deviant* (flute instead of a piano) could occur in the first, third or fourth tone of melodies (in 8% of the melodies). In addition, some timbre changes occurred in the ending tone (not analyzed here).
3. *Timing delay* (=100 ms silent gap) could occur in the first, second or third tone (in 8% of the melodies). In addition, some timing delays occurred in the ending tone (not analyzed here).

High-level changes

1. *Melody modulation* was presented as a pitch change of the third or fourth tone within the melody in 12% of them. It slightly changed the prevailing melody and endured until a new melody modulation was introduced.
2. *Rhythm modulation* (=reversal of the duration of two sequential tones) could occur in the second or third tone (in 7% of the melodies). There were thus two alternatives for rhythm modulation, either long tone was replaced by a short one (tone shortening) or short tone was replaced by a long tone (tone lengthening).
3. *Transposition* (one semitone up or down) could occur in the first triad (in 16% of melodies). After chord transposition the following melodies retained the converted key until a new chord transposition was introduced.

All high-level changes became the repeated form of the melody in the subsequent presentations in the so-called roving-standard fashion (Cowan et al., 1993). Thus, for example, after a modulation of the melody, the following melodies were repeated in the modulated form. In addition, all high-level changes were musically plausible, i.e., the resulting melody was in key and consonant, very similar to the other variants of the melody. Correspondingly, the rhythm modulations resulted in maintaining the beat of the repeated melodies, and the melody modulations were from 1 to 3 semitones such that they resulted in the new tones belonging to the original key.

### Participants

In total, there were 28 healthy adult participants involved in EEG recordings. Two of them were excluded from the EEG analyses, one based on noisy EEG and another one based on unspecific musical profile (this participant had some training in folk music but not advanced enough to justify being a member of that group, see below).

From the remaining 26 participants, 13 were active in learning and performing Finnish folk music. Their mean age was 26.7 yrs (range: 18–31 yrs, SD = 3.6), 10 of them were females. Currently they were either actively performing artists or students of the Sibelius Academy (Finnish university for music performance). They had started to play, on average, at the age of 7.8 years (range: 4–25 yrs, SD = 5.8 yrs). Their instrumental choices are listed in Table 1. Six of them named violin as the major instrument. The rest listed *kantele* (2; Finnish traditional instrument resembling Celtic harp), vocals (2), accordion, guitar, and wind instruments as their major instrument. In total, they named 21 instruments as their minor instruments. In this paper, these participants will be called *Folk musicians*. From the remaining 13 participants, most had not been involved in music lessons outside the school at all (*N* = 10) while some had some extracurricular music activities for 1–2 years prior to their puberty (*N* = 3). Their mean age was 25.1 yrs (range: 20–35 yrs, SD = 4.1), 11 of them were females. In this paper, they will be called *Non-musicians*.

**Table 1:** **Instrumental background of the Folk musicians**.

**Main instruments**	**N of musicians**	**Other instruments**
Violin	6	Piano
Folk wind kantele	2	Harmonium
Vocals	2	Mandolin
Accordion	1	Guitar
Accordion (2 rows)	1	Vocals
Guitar	1	Percussions
		Kantele
		Jouhikko
		Keyboards
		Nyckelharpa
		Accordion (2 rows)
		Estonian bagpipes
		Didgeridoo
		Viola
		Drums
		Saxophone
		Banjo
		Violin
		Bass guitar
		Bouzouki
		Double bass

### Experiment

#### Procedure

During the EEG recordings, the participants were sitting in a dimly lit EEG chamber in a comfortable chair. They were instructed to watch a silent nature documentary while stimuli were presented via headphones. The EEG recording was preceded by a 10 min session during which the participants were asked to listen to three self-selected music samples. These data will be reported elsewhere.

#### EEG recordings

The recordings were conducted in an acoustically and electromagnetically shielded room (Euroshield Ltd., Finland) of the Institute of Behavioural Sciences, University of Helsinki.

The EEG was recorded with BioSemi–system with a sampling rate of 4096 Hz by using a 64-electrode EEG-cap and six additional facial silver-chloride (Ag/AgCl) electrodes. They were inserted on the mastoids, on the tip of the nose, under right eye (for EOG monitoring) and two on EMG-related (electromyography) sites on left cheek and over the left eyebrow. The mean value of mastoid electrodes was used as a reference during the offline analyses. The EOG electrode was used to extract artifacts from the data due to eye blinks.

Hearing thresholds were determined by averaging five tests of just-audible sounds. A volume level of 60 dB HL over the individually determined hearing threshold was used. The sounds were presented via headphones.

### Data analysis

In the first step of preprocessing, data were referenced to the nose electrode, resampled to 256 Hz and, due to fast stimulation rate, filtered with 1-Hz high-pass cut-off. After this, data were divided into epochs from −100 ms prestimulus to 600 ms post-stimulus and individual data blocks were merged together as one dataset. As recommended by Onton and Makeig (2006) before independent component analysis (ICA), the removal of “non-stereotyped” or “paroxysmal” noise, associated with non-fixed (random) scalp projections, was obtained by rejecting epochs based on voltage amplitudes. This voltage rejection was done with a threshold of ±300 μV in most participants, of ±340 μV with four participants and of ±370 μV in one participant, depending on the quality of the data in those different experimental sessions. This initial cleaning of the largest artifacts based on voltage amplitudes was conducted to improve the performance of ICA decomposition, which is known to be optimal for separating only certain kinds of artifacts, and particularly those associated with fixed scalp projections. The final rejection of extra-encephalic artifacts was hence achieved with ICA, which was conducted with the *runica*-algorithm of the EEGLab-software. The independent components (IC’s) originating from eye blinks, ocular movements and other muscle artifacts were removed manually from the data of each individual subject. IC’s were identified based on their topographical location, frequency power spectrum, and temporal shape. After removing non-encephalic IC’s 25-Hz low-pass filtering was done and ±100 μV voltage rejection was applied to epochs to remove artifacts from ±100 to ±300 μV remaining in data. Finally, all the qualified data of each single subject was separately averaged according to the sound type (standard and the several different deviance types).

The ERP amplitudes were quantified by first determining the peak latencies from the grand-average difference waves separately for each sound change (deviant) as the largest peak between 100–300 ms at Fz for MMN and between 300 and 400 ms for P3a. After the peak definition, the amplitude values at the individual difference initiatives to use more ecologically valid music-like a 40-ms window centered at the ERP peak.

#### Statistical analysis

The group differences in the brain responses were tested with separate three-way mixed-model ANOVAs with MMN/P3a amplitudes as the dependent variables. Group (Folk/Non-musicians) was used as a between-subject factor and Laterality (left/midline/right) and Frontality (frontal/central/posterior) as within-subject factors. Factor Frontality consisted of the amplitude values obtained at three lines of electrodes: Frontal (F3, Fz, F4), Central (C3, Cz, C4), Posterior (P3, Pz, P4). Factor Laterality consisted of amplitude values as obtained at the Left (F3, C3, P3), Middle (Fz, Cz, Pz), and Right (F4, C4, P4). Subsequently, paired post hoc comparisons were conducted using Least Significant Different–test.

## Results

Using a novel melodic multi-feature paradigm with six sound changes (deviants) embedded within it, we show here that despite the complexity of the stimulation both Folk musicians and Non-musicians were able to neurally discriminate the changes from the regular melody continuation. This was evidenced by the presence of the MMN in both groups of participants to all other deviants except the rhythmic modulation in non-musicians (see Table 2).

**Table 2:** **Two-tailed t-tests for verifying the significance of the MMN amplitudes against the zero baseline**.

	**Mean**	**SD**	***T*-value**	**df**	***P*-value**
Folk musicians				
Mistuning	−2.7	1.1	−9.2	12	> 0.001
Timbre	−3.2	1.6	−7.3	12	> 0.001
Timing delay	−1.0/−1.2	1.0/2.2	−3.6/−1.9	12	= 0.003/= 0.08
Melody modulation	−1.9	1.3	−5.3	12	> 0.001
Rhythm shortening	−1.3	1.8	−2.4	12	= 0.03
Rhythm lengthening	−1.7	1.5	−4.1	12	= 0.001
Transposition	−0.7	1.0	−2.6	12	= 0.03
Non-musicians					
Mistuning	−0.7	0.9	−2.6	12	= 0.03
Timbre	−2.6	2.0	−4.6	12	= 0.001
Timing delay	−0.7/−1.4	1.4/1.5	−1.79/−3.6	12	= 0.09/= 0.004
Melody modulation	−1.9	1.2	−5.9	12	< 0.001
Rhythm shortening	−0.2	1.3	−0.6	12	=.6
Rhythm lengthening	−0.7	1.3	−2.0	12	= 0.07
Transposition	−0.8	0.8	−3.4	12	= 0.005

*For timing delay two tests were conducted since there were two distinct peaks observable*.

### Low-level changes

#### Mistuning

**Figure 2:**
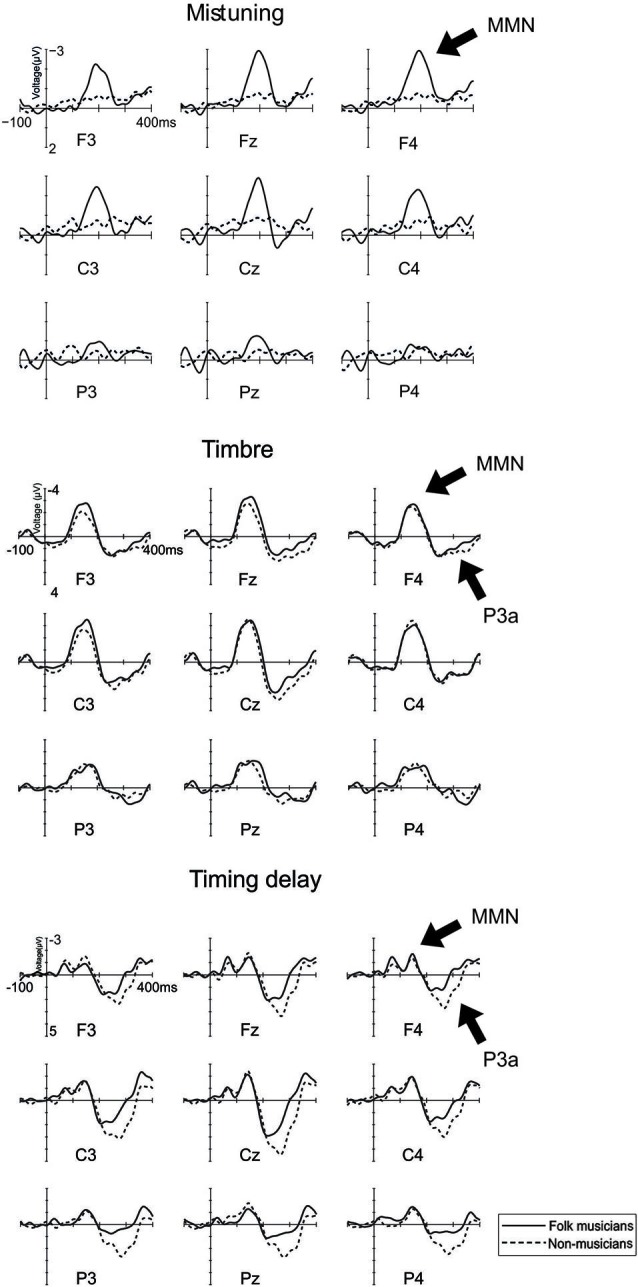
**Brain responses (ERP to deviant melody subtracted from the ERP evoked by the standard melody) in Folk musicians (continuous line) and Non-musicians (dashed line) to Mistuning, Timbre, and Timing delay**. These changes were introduced in the melody but they did not modulate the continuation of the melody.

As indicated in Figure 2, MMN to mistuning was significantly larger in Folk musicians than in Non-musicians (Group: *F*_(1,24)_ = 17.3, *p* < 0.0001). Moreover, it was frontally maximal and largest at the midline (Frontality; *F*_(2,48)_ = 24.1, *p* < 0.0001; Frontal MMN > Posterior MMN, *p* < 0.0001; Laterality; *F*_(2,48)_ = 5.1, *p* < 0.01; Midline MMN > Left MMN, *p* < 0.009, and Frontality × Laterality: *F*_(4,96)_ = 4.0, *p* = 0.01, ε = 0.7). Additionally, its distribution was modulated by musical expertise (Frontality × Group; *F*_(2,48)_ = 11.5, *p* < 0.0001), MMN being enhanced at the frontocentral electrodes in Folk musicians (*p* < 0.0001).

Folk musicians were highly sensitive towards mistuning as indicated by a significantly larger MMN when compared to that of Non-musicians. Additionally about half of the deviants initiated further evaluation and attentional processing of the participants as evidenced by the P3a elicitation (Table 3). These and other results are further specified below, first for low-level changes and thereafter for high-level changes.

**Table 3:** **Two-tailed t-tests for verifying the significance of the P3a amplitudes against the zero baseline**.

	**Mean**	**SD**	***T*-value**	**df**	***P*-value**
Folk musicians					
Timbre	1.4	1.8	2.8	12	= 0.02
Timing delay	1.8	1.5	4.2	12	= 0.001
Melody modulation	0.3	1.0	1.0	12	= 0.4
Rhythm lengthening	0.6	1.3	1.7	12	= 0.1
Transposition	1.5	0.9	5.8	12	< 0.001
Non-musicians					
Timbre	1.9	1.7	4.1	12	= 0.001
Timing delay	3.0	1.5	7.2	12	< 0.001
Melody modulation	−0.3	1.1	−1.0	12	=.3
Rhythm lengthening	0.2	1.0	0.8	12	= 0.4
Transposition	0.6	0.8	2.7	12	= 0.02

*T-tests were not conducted for mistuning and rhythm shortening since no P3a peaks were visible from the grand-average curves*.

#### Timbre

MMN to timbre change was maximal at mid-central electrodes (Frontality; *F*_(2,48)_ = 16.7, *p* < 0.0001, ε = 0.6; central MMN > frontal and posterior MMN, *p* < 0.006; Laterality: *F*_(2,48)_ = 9.1, *p* < 0.001; Midline MMN > Left and Right MMN, *p* < 0.004). Additionally, its distribution was modulated by the musical expertise (Frontality × Laterality × Group, *F*_(4,96)_ = 5.2, *p* = 0.001). According to *post-hoc* tests, in Folk musicians, the MMN over the left hemisphere and midline electrodes was larger at frontal and central electrodes than at posterior ones (*p* < 0.04). Over their right hemisphere, the MMN was larger at central than at posterior electrodes (*p* = 0.001). In contrast, in Non-musicians, the left central MMN was larger than posterior MMN (*p* = 0.03), mid-central MMN was larger than the frontal and posterior MMN (*p* < 0.02 in both comparisons), and the right central MMN was larger than the frontal MMN which, in turn, was larger than the posterior MMN (*p* < 0.02 in all comparisons).

P3a to timbre change was frontally maximal and largest at the midline (Frontality: *F*_(2,48)_ = 19.9, *p* < 0.0001; frontal and central P3a being larger than posterior P3a, *p* < 0.002 for all; Laterality: *F*_(2,48)_ = 17.5, *p* < 0.0001; midline P3a being larger than both left P3a and right P3a, *p* < 0.0001; Laterality × Frontality: *F*_(4,96)_ = 6.7, *p* < 0.0001). However, musical expertise did not significantly modulate the P3a amplitude or its distribution above the scalp.

#### Timing delay

Timing delay elicited two subsequent MMN responses. The first one was frontocentrally maximal (Frontality; *F*_(2,48)_ = 9.0, *p* = 0.004, ε = 0.6, frontal and central MMN larger than posterior MMN, *p* < 0.009 for all). Additionally, its distribution was modulated by the musical expertise (Laterality × Group, *F*_(2,48)_ = 6.5, *p* = 0.003; Frontality × Laterality × Group, *F*_(4,96)_ = 4.5, *p* = 0.002), with a tendency for a larger MMN in Folk musicians over Non-musicians at the left posterior electrode site (*p* = 0.08). Moreover, in Folk musicians the MMN over the left hemisphere was largest at frontal and central electrodes whereas it was minimal at the posterior site (*p* = 0.006). At midline electrodes their MMN was largest frontally and smallest posteriorly (*p* < 0.02). Over the right hemisphere there were no indications of frontality (*p* > 0.05). In Non-musicians the scalp distribution followed a different pattern: they also showed the MMN over the left hemisphere which was largest frontally and smallest posteriorly (*p* < 0.03). Over the midline electrodes there were no indications of frontality and, finally, over their right hemisphere the MMN was largest centrally, smaller at the frontal electrodes and smallest at the posterior electrodes (*p* < 0.05).

The second one was maximal at central electrodes (Frontality: *F*_(2,48)_ = 4.5, *p* = 0.04, ε = 0.7; central MMN being larger than the frontal MMN and posterior MMN, *p* = 0.001 for all; Laterality: *F*_(2,48)_ = 4.7, *p* = 0.01; midline MMN being larger than the left MMN, *p* = 0.005). Its distribution was modulated by musical expertise (Frontality × Laterality × Group: *F*_(4,96)_ = 3.3, *p* = 0.01), resulting from the varying pattern of frontality between Folk musicians and Non-musicians. Folk musicians had centrally and posteriorly larger MMN than their frontal MMN at left and midline sites (*p* < 0.05 for all). Moreover, their MMN over the right hemisphere had no indication of frontality (*p* > 0.05). Non-musicians showed a broadly distributed MMN at left sites and a central MMN which was larger than their frontal MMN at midline sites (*p* = 0.01) and a central MMN which was larger than their posterior MMN at right sites (*p* = 0.003).

The MMN to timing delay was followed by a P3a that was maximal at central electrodes (Frontality: *F*_(2,48)_ = 22.3, *p* < 0.0001; central P3a was larger than frontal P3a which, in turn, was larger than Posterior P3a, *p* < 0.02 for all; Laterality: *F*_(2,48)_ = 22.4, *p* < 0.0001; midline P3a being larger than left and right P3a, *p* < 0.0001). Additionally, its scalp distribution was modulated by musical expertise (Frontality × Laterality × Group: *F*_(4,96)_ = 4.1, *p* = 0.004), due to the larger P3a in Non-musicians compared to Folk musicians over the right frontal region (*p* = 0.01) and left posterior region (*p* < 0.05).

### High-level changes

#### Melody modulation

**Figure 3:**
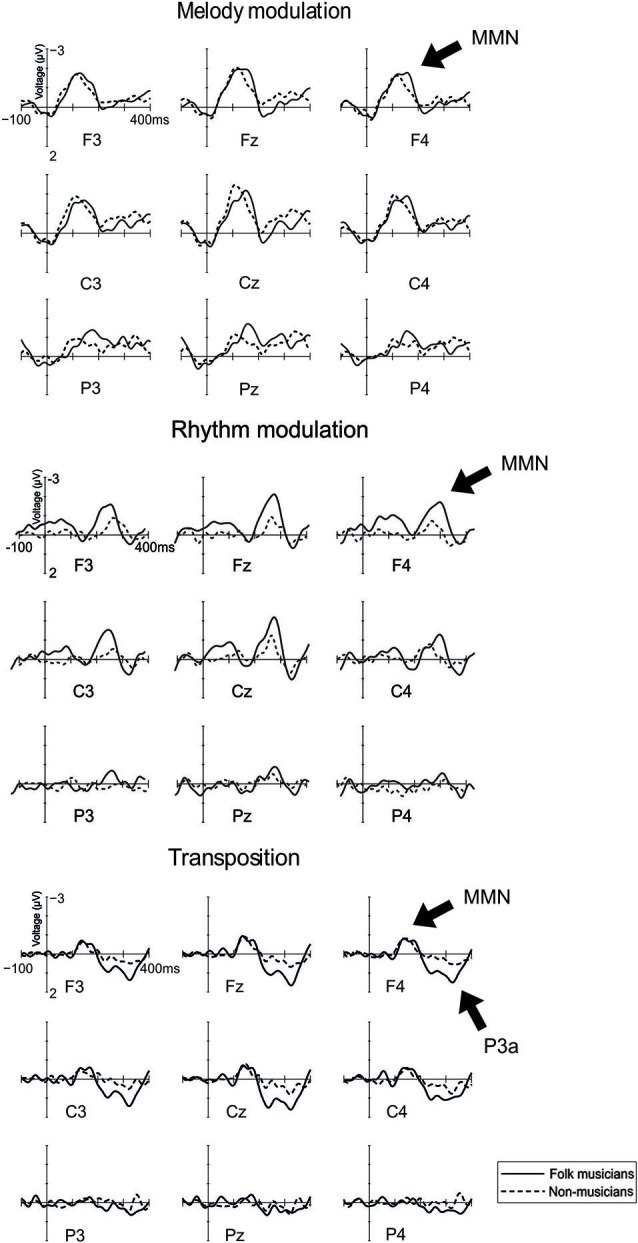
**Brain responses to Rhythm modulation, Melody modulation, and Transposition**. These changes were introduced in the melody and they modulated its continuation.

MMN to melody modulation was maximal at mid-central electrodes (see Figure 3; Frontality: *F*_(2,48)_ = 21.1, *p* < 0.0001; frontal and central MMN being larger than posterior MMN, *p* < 0.001; Laterality: *F*_(2,48)_ = 16.4, *p* < 0.0001; midline MMN being larger than left and right MMN, *p* < 0.0001). Additionally, its distribution was modulated by musical expertise (Laterality × Group: *F*_(2,48)_ = 3.9, *p* = 0.03). This was caused by the MMN amplitudes which did not differ from each other between the left and right site electrodes but which were largest at the midline region (*p* < 0.05) for Folk musicians compared to other electrode sites. For Non-musicians, the MMN was larger at the left electrodes compared to the right ones (*p* = 0.02). Additionally, the left and right MMNs were smaller than the MMN at the midline (*p* < 0.006 for both comparisons). Also an interaction between Laterality and Frontality was observed (*F*_(4,96)_ = 5.0, *p* = 0.001), deriving from larger MMN at the frontal and central electrodes than at the posterior and lateral electrodes (*p* < 0.01 in all comparisons).

#### Rhythm modulation: shortening of a long tone

MMN to rhythm modulation of tone shortening was maximal at frontocentral midline electrodes (Frontality: *F*_(2,48)_ = 7.5, *p* = 0.007, ε = 0.6; frontal and central MMN being larger than posterior MMN, *p* < 0.02; Laterality: *F*_(2,48)_ = 6.4, *p* = 0.004; midline MMN larger than left and right MMN, *p* < 0.002).

#### Rhythm modulation: lengthening of a short tone

MMN to rhythm modulation of tone lengthening was frontocentrally maximal (Frontality: *F*_(2,48)_ = 4.0, *p* = 0.04, ε = 0.8; Frontal and Central MMN > Posterior MMN) and modulated by musical expertise (Frontality × Group: *F*_(2,48)_ = 7.4, *p* = 0.002). This resulted from larger MMN above the frontal and central electrodes (vs. posterior ones) in Folk musicians only (*p* < 0.002 for both) and small uniformly distributed MMN in Non-musicians (*p* > 0.05 in all comparisons).

#### Transposition

In both groups, MMN to transposition was maximal at the frontal electrodes (Frontality; *F*_(2,48)_ = 12.1, *p* < 0.0001, ε = 0.7; frontal and central MMN being larger than posterior MMN, *p* < 0.001). P3a to transposition was maximal at frontocentral midline electrodes (Frontality: *F*_(2,48)_ = 25.6, *p* < 0.0001, ε = 0.8; frontal and central P3a larger than posterior P3a, *p* < 0.0001; Laterality: *F*_(2,48)_ = 3.4, *p* = 0.04; midline P3a larger than left and right P3a, *p* < 0.03). It was modulated by musical expertise (Frontality × Group: *F*_(2,48)_ = 7.5, *p* = 0.001; Frontality × Laterality × Group: *F*_(4,96)_ = 3.0, *p* = 0.02): In *post-hoc* analyses, Folk musicians showed a larger P3a overall at frontal electrodes (*p* = 0.008) and at the left central electrode (*p* = 0.03) when compared with Non-musicians.

## Discussion

In the present study, we use the novel melodic multi-feature paradigm for investigating the brain basis of early musical sound encoding using the MMN and P3a components of the auditory ERPs. We composed a short melody with several changes embedded in it (Figure 1). “Low-level” changes in timbre, tuning, and timing interspersed the regular melody content, but were not included in the continued melody. In contrast, “high-level” changes in melody contour, rhythm, and key modified the continuation of the melody. The melody was presented in a looped fashion without any pauses between the subsequent melodies. Half of our participants were professional musicians with professional-level expertise in current Finnish folk music. According to the present results, the training in folk music is especially reflected in the brain encoding of pitch, as evidenced by larger MMN for mistuning observed in Folk musicians than in Non-musicians. Moreover, all changes evoked MMN or P3a responses with different scalp distributions in Folk musicians than in Non-musicians implying that the brain dynamics can be relatively broadly modulated by the music activities. Importantly, we also show here that this novel melodic multi-feature paradigm provides an excellent tool for revealing the auditory neurocognitive profile of musically non-trained adults.

To specify the results further, the MMN and P3a responses were enhanced or generated with slightly different brain architectures in Folk musicians and in non-musicians. These effects of musical expertise were most obvious for pitch information in terms of enhanced MMN specifically to mistuning in Folk musicians. This finding coincides and upgrades our previous results which indicated that Folk musicians encode chord cadences in a very unique manner (Brattico et al., 2013): in addition to generally enhanced early anterior negativity (ERAN) around 200 ms, Folk musicians had subsequent late ERAN and P3a responses as a function of the given inappropriate chord position. More specifically, Folk musicians showed a subsequent P3a only to the strongly-violating ending chord and a late ERAN in response to the milder chord violation at the fifth position (where it violated less the expectations of the listeners). Since in Brattico et al. (2013) the ERAN was recorded in a semiattentive paradigm when the participants were instructed to detect timbre-deviant chords (but not inappropriate chord functions), those results, however, leave it open as to whether the auditory sensitivity of Folk musicians exists already at the pre-attentive stage of processing. Even if the current study does not allow us to completely rule out the involvement of involuntary attentional functions either (especially for deviances in timbre, timing, and transposition as they were followed by P3a responses) particularly mistuned sounds seem to be encoded without the triggering of involuntary attention. This further strengthens the conclusion about a parameter (pitch, harmony) specific enhancement of sound processing in Folk musicians.

Notably, pitch is a pivotal dimension in Finnish folk music. Similarly to folk music in other parts of the world, Finnish folk music stresses the importance of improvisation and variation of a motif by adding grace notes or various kinds of auxiliary notes (Saha, 1996). These pitch-based features are partly a consequence of the independence of folk music from musical scores: most folk tunes are transmitted from generation to generation through performance practice and memory rather than written scores. Moreover, Finnish folk music is often monophonic or heterophonic, with contemporary variations of the same lines of music played according to the idiomatic characteristics associated with the performance of a particular instrument (Asplund et al., 2006). For instance, music played by the *kantele* is repeated and varied particularly in the melody and harmony dimensions (Saha, 1996). Hence, our findings of pitch-specific enhancements of ERP responses contribute to the growing literature on auditory neuroplasticity specific to the idiosyncratic practices of a musical style (see below).

Previously, parameter-specific pre-attentive enhancement of sound processing has been evidenced among classical musicians for timbre (trumpet *vs*. violin players) (Pantev et al., 2001) and spatial sound origin (conductors *vs*. pianists) (Nager et al., 2003). It was also shown for pitch (Koelsch et al., 1999) in violin players without being generalizable for other instrumentalists (Tervaniemi et al., 2005). Correspondingly, it was shown for spatial sound origin and intensity in rock musicians (Tervaniemi et al., 2006) and, among other sound features, also for rhythmic changes and pitch glides in jazz musicians (Vuust et al., 2005, 2012). Together with the current MMN data and previously introduced ERAN evidence (Brattico et al., 2013), we are tempted to conclude that the sound parameters which are of most importance in a given musical genre or which became most familiar during the training history (e.g., timbre of one’s own instrument) become particularly sensitively processed during the course of the musicians’ training history. This line of reasoning is supported by the current finding with regard to the rhythm MMN which, in contrast to pitch MMN, was enhanced in Folk musicians when compared with non-musicians only when tones were lengthened but not when they were shortened. In other words, rhythmic modulation was not encoded by Folk musicians in great detail. This is not a surprise when keeping in mind the special characteristics of Finnish folk music as described above. In contrast, rhythm MMN did differentiate jazz musicians and non-musicians in a pioneering MMNm study (Vuust et al., 2005). In jazz, very fine-grained modulations in the timing of the performance carry high importance in expressivity of music.

Yet, without a large cross-sectional study systematically comparing musicians from several genres (e.g., classical, jazz, rock, folk) using a same stimulation paradigm such a conclusion should be considered only as a tentative one. By such a laborious procedure one would obtain reliable information only about the current functionality of the auditory encoding in these musicians.

It is noteworthy that even then the outcome of such a cross-sectional study conducted in adult musicians would not reveal whether the resulting auditory profile reflects the auditory encoding abilities as they were prior to commencement of play, or their maturation, and enhanced development due to training in music, or their combination. Therefore, there is an urgent need for longitudinal projects on children who are either involved in musical programs (musically active children) or in other training programs of comparative intensity and content but without a musical aspect (control children). Only those can reveal the relationship between the original auditory encoding accuracy and its further development, for instance, in terms of sound parameters that are unequally important in musical genres and instrument families. First of study along these lines was recently conducted by Putkinen et al. (2014). According to those results, the children actively training to play classical music displayed larger MMNs than control children for the melody modulations by the age 13 and for the rhythm modulations, timbre deviants and slightly mistuned tones already at the age of 11. At the onset of the study when children were 9 years old, there were no group differences. Thus, the current paradigm is also sensitive with regard to the development of the neural sound discrimination during musical training.

Methodologically, the current paradigm provides a novel means for determining the integrity of the auditory neurocognition simultaneously for six acoustical and musical parameters. It thus updates the tradition initiated by the multi-feature paradigm, which used isolated sinusoidal or speech-sound stimulation (Näätänen et al., 2004; Pakarinen et al., 2007; Partanen et al., 2011). Recently, the multi-feature paradigm was also successfully extended into fast musical MMN paradigm using instrumental sounds in a harmonic accompaniment setting (Vuust et al., 2011, 2012). The current paradigm is an upgrade of that since it includes both low- and high-level deviances, which either preserve or modify the melody. In parallel, one should not ignore previous initiatives to use more ecologically valid music-like stimuli in MMN studies (see, e.g., Tervaniemi et al., 2001; Fujioka et al., 2004, 2005; van Zuijen et al., 2004; Brattico et al., 2006; Virtala et al., 2012, 2013). Even if those included a more limited number of deviances than the more recent multi-feature paradigms, they were able to reveal novel information regarding preattentive encoding of musically relevant sound-attributes both in musically trained and in non-trained groups of participants.

Highly promisingly, we show here that despite the complexity of the melodic multi-feature paradigm, the MMN could also be observed at the group level in non-musicians for all deviances except rhythm modulation. Moreover, the paradigm has been successfully used also in child recordings (Putkinen et al., 2014). Thus, in the future, auditory profiles of child and adult participants can be probed during the whole life span with various multi-features paradigms using sinusoidal, linguistic, and musical stimulation entities approaching natural sound environments as present in everyday life.

## Conflict of interest statement

The authors declare that the research was conducted in the absence of any commercial or financial relationships that could be construed as a potential conflict of interest.
